# “Are you OK doctor?” An expanded health belief model exploration of doctors’ experiences and perspectives of on-shift health behaviour

**DOI:** 10.1080/17482631.2024.2388795

**Published:** 2024-08-05

**Authors:** Kirsty L. Hodgson, Daniel J. Lamport, Allán Laville

**Affiliations:** School of Psychology & Clinical Language Sciences, The University of Reading, Reading, UK

**Keywords:** Health belief model, health behaviour, doctors, thematic analysis, healthcare, United Kingdom, COVID-19

## Abstract

**Purpose:**

Understanding doctors’ health beliefs is essential for developing effective and competent healthcare practices that benefit doctors and their patients. This study aimed to qualitatively explore doctors’ perceptions of on-shift health-protective behaviours and their perceived effects on competence.

**Methods:**

The research applied theoretically driven Expanded Health Belief Model (EHBM) enquiry methods to explore beliefs and experiences through an occupational context survey, 14 individual depth interviews, and two focus groups. Semantic and deductive themes associated with EHBM domains were examined, and an inductive thematic analysis of the interviews was conducted.

**Results:**

Doctors’ beliefs were strongly imbued by their perceived identity within the systemic context; they expressed impaired self-efficacy in reacting to their health needs on shift, and several disclosed harm to themselves and patients. Dominant themes included the psychosocial effects of the systemic culture and the influence of the situational occupational context in impacting health-protective behavioural action. The context and implications of experiences during the COVID-19 pandemic are discussed.

**Conclusions:**

This study presents key belief-oriented factors influencing doctors’ health-protective behaviour at work and its implications for competent practice. Further doctor-led guidance on focus points for evidence-based theoretically driven health improvement solutions is provided regarding operational practice, formulating policies, developing interventions and further research.

## Background

The medical profession is inherently demanding, and competence depends on the doctor’s optimal state of well-being. Therefore, understanding doctors’ on-shift health needs, addressing factors that impair well-being, and the beliefs mediating doctors’ health behaviours is an area of research that benefits doctors and patient care. Resultant research-driven strategies may mitigate occupational hazards and promote a supportive workplace, ensuring a resilient and sustainable medical workforce.

The National Health Service (NHS) in the United Kingdom (UK) faces escalating demands, which increases in budget have not fully accommodated (Anadaciva, 2023), impeding optimal patient care and maintenance of safe working conditions (Amalberti & Vincent, 2020). The novel coronavirus (COVID-19) pandemic (World Health Organization, 2021) impacted doctors by creating unprecedented exigencies encompassing professional, personal, and systemic dimensions (Buchbinder et al., 2023; Cubitt et al., 2021). This impact was particularly severe in emergency departments, primary and acute care (Sanford et al., 2022). Doctors’ dissatisfaction with salaries, work–life balance, staffing levels and job dissatisfaction have led to a series of strikes, further aggravating the crisis (British Medical Association, 2023; Irving et al., 2024). Doctors’ mental and physical health have become increasingly salient concerns, necessitating ongoing research, policy consideration, and intervention to safeguard their well-being and the quality of healthcare.

Although the NHS has unique nuances and effects on staff well-being, the pressures are not all unique to the U.K. In a national study of burnout in a large sample (*N* = 5197) of American doctors and a probability-based sample of the general population for comparison, Shanafelt et al. (2019) measured burnout and satisfaction with work–life balance, finding high levels of work-related distress with associated risks for patient care and medical errors (Tawfik et al., 2018). International studies on medical error related to human factors have highlighted a need to better understand well-being-related predictors and the mediating risk disparities within medical professions (McKinley et al., 2020; Trockel et al., 2020; Yan et al., 2023). Therefore, an improved understanding of the beliefs and motivators of clinician well-being and the effects of associated patient care is globally relevant. Examining the challenges and implications of NHS doctors’ well-being at work can potentially yield insights that may affect the diverse health service workforce and have implications for broader global health systems.

### Theoretical framework

Despite growing recognition of the importance of “fitness-to-practice”, there is limited research understanding of the beliefs that mediate doctors on-shift health behaviours. Understanding beliefs that motivate decision-making and determine mechanisms of behaviour may serve as change objectives to inform health-promotion interventions (Downing-Matibag & Geisinger, 2009). The Health Belief Model (HBM) was originally developed by Rosenstock (1966) to understand beliefs and cognitive-appraisal mechanisms associated with intention to change (Munro et al., 2007). The HBM contends that beliefs predict actions and has been applied internationally as an effective psychological framework to aid change processing, inform health improvement interventions, and predict treatment adherence (Azadi et al., 2021; Kudo et al., 2011). The expanded HBM framework (EHBM) developed by Rosenstock et al. (1988) encompassed the key mediating and modifying factors of demographic variables and psychological characteristics in addition to the original predictive motivating domains: perceived susceptibility, perceived severity, perceived benefits, perceived barriers, and the behavioural activation domains of cues-to-action and action. This expanded theoretical framework was selected a priori for this study as its multidimensionality corresponded with the qualitative aims of this research. Previous research has not examined the on-shift health behaviour of doctors using the EHBM. Therefore, qualitative research approaches that accommodate the unique circumstances of medical professionals affords insight into appropriate interventions for promoting well-being, enhancing competence, and improving patient care.

### Study objectives

This study aimed to explore doctors’ beliefs regarding on-shift health-protective behaviours. The primary research question addressed was, “*What are doctors” perceptions of health-protective behaviors during a working shift?*’ The secondary research question was, “*What effects do doctors believe practicing health-protective behaviours during their shifts have on their professional competence?*”

## Methods

### Design

Theoretically driven qualitative inquiry was employed to explore doctors’ beliefs and experiences. The rationale for employing the EHBM lies in its capacity to delve into the nuanced aspects of beliefs, and contextual factors influencing health-related behaviours. Qualitative individual depth interviews (IDIs) and focus group interviews (FGIs), were enhanced interpretation of doctors’ construction of their health beliefs individually and how these manifested in their behaviour within a group to examine dynamic social deliberations and personality-sensitive dynamics.

This study recognized the duty of care to participants and institutions affiliated with this research to maintain integrity and respectful practice with a strong awareness of moral rights, legal compliance, upholding scientific standards and avoiding personal or professional harm. This study adhered to the guidelines dictated by The British Psychological Society’s Code of Human Research Ethics (Oates et al., 2021). Detailed ethical considerations may be found in the research protocol in *Supplementary File 1*.

### Participant recruitment

Following ethical approval granted by the institutional review board at the PCLS School Research Ethics Committee (SREC) at the University of Reading (approval no. 2022129AL), this study purposively recruited a sample that sought diverse information-rich interviews with doctors at various stages and occupational contexts. Therefore, participants unknown to the researchers were invited through social media circulation methods on doctor support networks via a recruitment advert with a registration form for a telephone briefing. Inclusion criteria were doctors employed by the NHS in the UK. Excluded from participation were retired doctors, those not currently working or working in settings outside the NHS.

Guiding principles to determine sample sizes were led by the study purpose and parameters of meaning saturation proposed by Hennink et al. (2017), who found that a richly textured understanding of thematic issues may be identified among 16–24 interviewees. Focused on capturing the meaning of codes versus the prevalence, the anticipated recruitment was 25 participants (to allow for non-completion of all study requirements). Thirty-one interested doctors completed the screening survey; one did not fulfil the NHS employment status inclusion criteria, and two provided insufficient contact details. Twenty-eight doctors who met the inclusion criteria were invited to participate in this study, of whom 24 doctors completed the study survey and either an IDI (*n* = 14) or participated in one of two FGIs (*n* = 4, *n* = 6), all were included in analysis. As this was at the upper range proposed by Hennink et al. (2017), this sample was considered sufficient for a qualitative enquiry of this nature to achieve the depth and meaning saturation parameters required to address the research questions in this purposive sample according to comparable qualitative health research (Vasileiou et al., 2018). The researchers’ reflective assessment and evaluation of the doctor’s thematic narratives indicated high information power, which may be considered a more reliable benchmark in qualitative enquiry (Malterud et al., 2016). Participants were allocated anonymized identifier codes; for doctors who participated in interviews, these appear as (i1–14), for focus groups; FGI-1 (f1.1–4) and FGI-2 (f2.1–6).

### Procedure

Following explicit informed written consent, participant-doctors completed an online contextual survey, followed by an IDI or a FGI that included fewer than eight participants (as advocated by Kruger & Casey, 2014). All interviews were conducted, transcribed, and recorded using Microsoft Teams videotelephony online conferencing software.

#### Electronic survey

A brief electronic survey using licenced academic research survey software “Online Surveys” (OS) was employed. The brief survey comprised sociodemographic questions and questions pertaining to occupational context and role. The interview schedule was designed to exclude specific enquiries regarding demographic variables (particularly in the focus group context) to support doctors in pre-considering the specificity of potentially identifiable information they felt comfortable disclosing. This survey included the demographic variables of age, sex, gender, ethnicity, and faith/religion alongside occupational role to examine the diverse characteristics that may influence health beliefs in doctors. The self-identified contextual demography from the survey was used to situate each doctor’s orientation and perspective alongside any participant-led interview disclosures to analyse the demographic themes within the EHBM.

#### Interview protocol development

The interview protocol and schedule (*Supplementary file 1*) was generated to guide an outline for exploration. To explore beliefs, indicative interview questions and probes were generated by mapping enquiries onto the EHBM domains to guide the structure and exploration. The principal early criticisms of the HBM (Rosenstock, 1966) was its exclusion of self-efficacy and cues to action (Hounton et al., 2005); therefore, these have since been incorporated (e.g., Gillibrand & Stevenson, 2006) and were also adopted domains in this study. Semi-structured questions in the interview schedule were designed to harvest meaningful data by encouraging doctors to openly reflect on their experience (Joffe, 2011). Although interviews were designed to reflect the EHBM, they were not restricted to the model’s domains, doctors were given ample time to share any associated experiences, which provided a broader exploration of additional mediating factors.

### Transcription

A transcription and anonymization protocol informed by recommendations by Clarke and Braun (2013; Braun and Clarke, 2022) and Saunders et al. (2015) was developed and followed to ensure accuracy of the data corpus and adopt a transparent, reproducible, and rigorous approach to the screening of the qualitative data and preserve the integrity of the responses and communication of salient themes (*Supplementary file 1*). Orthographic transcription methods were applied to present a complete and clear rendering of the interviews.

### Analysis

Quantitative data from the survey was explored using descriptive statistics to contextualize the interviews. Open-question free-text survey responses were analysed using thematic analysis to explore experiences and identify common themes and disparate experiences of on-shift health behaviours. An open survey question requiring doctors to list all on-shift health protective behaviours enabled the development of an occupationally apposite coding structure that was also applied to transcript analysis for further examination.

Thematic analysis methods as described by Braun and Clarke (2022) were applied to the interview transcripts as an iterative, mixed deductive-inductive approach whereby the analysis had a predefined analytical frame but remained open to emergent patterns and themes (Robson & McCartan, 2014). A hybrid process advocated by Fereday and Muir-Cochrane (2006) primarily focused on semantic themes, but also contextualized how participants constructed their expressed views and their relative importance at a latent level. These analysis methods were applied to address the dual aim of both theory mapping and to identify actionable leverage for intervention, as it was sufficiently flexible to be compatible with the varied analytical frameworks employed to examine the associated EHBM domains whilst at the same time providing a clear structure for systematic analysis. Analysis was conducted in Excel to aid organization iteratively into initial overarching themes, sub-themes, and categories within the deductive sub-themes. The EHBM domains were applied in a structured data coding framework method (Gale et al., 2013) determined by the deductive theory-driven codes. However, the secondary identification of inductive within-domain analysis and identification of overarching themes was also analysed. Where appropriate, additional inductive themes were added, for example, from the open survey questions and interviews, the preliminary reflexive analysis identified that systemic factors were not covered by the structured framework based on the EHBM domains; this was, therefore, added to the framework as an inductively driven theme for coding. _[T]_Themes, _[ST]_sub-themes, and _[FT]_further themes and specified analysis categories associated with the research aims were then collated into one framework to create an overarching conceptualization of the material.

Following interview transcription, primary analysis through active recusant immersion techniques commenced. Braun and Clarke (2019) “using the codes as building blocks” (p.855) approach was employed to find commonality among the codes and shared patterned meaning across the dataset. Recorded analysis reflections were guided by Braun and Clarke’s (2012) “suggested questions” and used to inform further structured data extraction and candidate coding. Initial themes were generated from the preliminary codes and established in an analysis file.

The contextualist approach applied reflexive thematic analysis alongside the survey data. This approach enabled theoretical flexibility and compatibility with both essentialist and constructionist paradigms (Braun & Clarke, 2022) to address the research aim of this study. The “six phases” of thematic analysis (Braun & Clarke, 2021) were conducted as an iterative process through the methods and were further detailed in the reflexive analysis protocol (*Supplementary file 1*).

### Analytic interpretation, researcher positionality and epistemological approach

All researchers engaged in an ongoing reflexive triangulation dialogue regarding the recursive interpretive analytic process and reviewed final themes and codes to enhance concordance and confirmability (Nowell et al., 2017). Through assessment of heterogeneity and homogeneity, themes were reviewed and refined to ensure their “central organizing concept” Braun and Clarke (2019). Linguistics were assessed for the objectivity of interpretation, reflectivity of terms, and labelling of codes and themes. Findings were then contextualized within the literature and extracted corroborated quotes from the interviews that captured key analytic essence were embedded to illustrate the analytic narrative.

This study adopted a critical realist epistemology, accepting that reality is complex and nuanced and acknowledging the role of subjectivity and interpretation. The critical qualitative interpretation approach warranted by this research aims to present subjective experiences while analysing the nuanced value and belief mechanisms associated with decisional balance and health behaviour. This approach values the researcher’s subjectivity as a resource (Braun & Clarke, 2019) in extracting meaning from the interviews. Although this research sought to give voice using a critical realist epistemological approach, authors acknowledge that all analysis is vulnerable to interpretative variability. Perspectives can be interpreted “based on the values, standpoints, and positions of the author” (Daly, 2007, p. 33). Therefore, this study acknowledges the interpretative lens that may have influenced analysis in a statement outlining the researchers’ positionality, aligning with an approach that prioritized reflexivity, sensitivity and research integrity as advocated by Muthanna and Alduais (2023). We declare the following reflexive positionality statement:

The researchers associated with this study acknowledge the lived experiences and the interpretative lens that may have influenced the analytic framework. Three researchers from the psychology department at the University of Reading in the UK collaboratively designed the study methodology, survey, and interview schedule. All authors have a non-medical professional background, though two (AL, KH) have had professional experience in employment within the NHS as psychologists. All authors have engaged with doctors personally and professionally within their professional lifespan. DL and KH have experience undertaking mixed-methods research. However, AL held substantial experience in thematic analysis and guided the qualitative analysis protocols in this study. Interviews were undertaken by KH in alignment with the agreed interview schedule, with regular consultation from AL and DL. KH has a professional background in applied clinical psychology and health psychology; her experiences as a recipient of health services and carer to health-compromised relatives, alongside her understanding of doctor colleagues, have shaped her motivation to explore this research area for the benefit of doctors and any associated impact on patient care. Researchers collaboratively generated initial codes by iterative refining, comparing, re-coding, grouping, defining, and labelling codes. There was no disagreement regarding the reflectiveness of the identified themes. However, their grouping within thematic mapping was reorganized through negotiation, and the re-reviewing of the transcripts recursively process continued until there were no proposed revisions. All authors were engaged in interpreting findings and adopting a critical introspective analytic process. However, this study offers one interpretation of the doctors’ experiences in this study, and it is essential to consider the researcher’s relationship to the research topic. The findings offer one interpretation of the doctors’ experiences in this study, and “contextualizing the researcher in relation to the topic or research being conducted is essential because it could expose limitations of the results of the research and how it should be interpreted” (Woo, 2022, p. 291).

## Results

The survey provided foundational background information on the doctors’ occupational orientation and contextual demography. Table 1 presents detailed self-identified contextual demography for all participants.

**Table 1.** Contextual demography.

Contextual Demography for All Participants						
Interview Mode	ID	Role	Sex	Age	Ethnicity	Faith/Religion
IDIs	I1	Consultant	Male	64	White British	Christian
	I2	Consultant	Female	45	Asian	None
	I3	Junior Doctor	Female	26	White Scottish	None
	I4	Consultant	Male	46	White British	Christian
	I5	Junior Doctor	Female	33	White English	Christian
	I6	Consultant	Male	42	Indian	Sikh
	I7	Junior Doctor	Male	34	White British & Australian	Christian
	I8	Junior Doctor	Female	31	Chinese	Christian
	I9	Junior Doctor	Male	37	White British	Atheist
	I10	Consultant	Female	61	White British	Christian
	I11	Junior Doctor	Male	27	White British	None
	I12	Junior Doctor	Male	32	Chinese	None
	I13	Junior Doctor	Male	35	White English	None
	I14	Junior Doctor	Female	24	White American	Spiritual
FGI 1	F1.1	Consultant	Male	52	Black English	Christian
	F1.2	Junior Doctor	Male	38	White English	None
	F1.3	Junior Doctor	Female	34	White English	None
	F1.4	Consultant	Female	64	White English	Christian
FGI 2	F2.1	Junior Doctor	Female	33	Chinese	None
	F2.2	Consultant	Male	63	White British	None
	F2.3	Junior Doctor	Male	25	White British	Buddhist
	F2.4	Junior Doctor	Female	25	White British	None
	F2.5	Junior Doctor	Female	23	White British	None
	F2.6	Consultant	Female	47	White Irish	Catholic

*Participants self-identified their characteristics; therefore, doctors’ descriptors are based on their described level of specificity.

**Figure 1.**
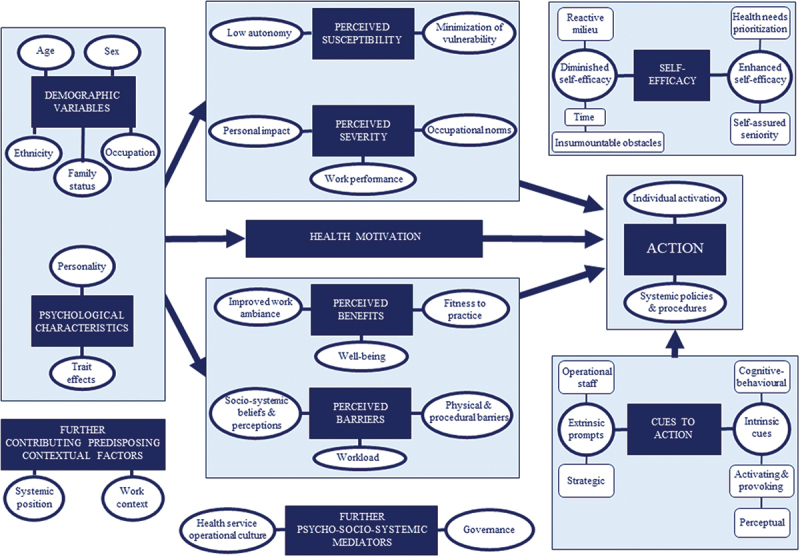
Thematic mapping of the EHBM.

To examine the role of belief-oriented mediators of doctors’ on-shift health behaviour, the EHBM-generated interview schedule probed responses and provided an initial deductive thematic analysis framework. Relative to the findings, each domain was examined for associated elicited beliefs and thematically mapped in the modified framework (Figure 1).

### Modifying demographic variables

Though the interview schedule included no specific enquiry regarding demographic variables, participant-led disclosures were deductively coded. Self-identified contextual demography from the survey was used to situate each doctor’s orientation and perspective. In addition to the prevalent overarching consistent themes, individual differences offered noteworthy insight into demographic considerations. Key modifying demographic variables included the following themes.

This study included a culturally diverse demographic with various religious and faith-based orientations, which was representative of the diverse NHS workforce (Niven et al., 2019). Ethnicity impacted identity, with subthemes of belonging, values, and beliefs, in addition to integration and inclusion for those trying to adapt to cultural norms and expectations for those new to the UK. There was substantial reflection about how working in different countries impacted their work demands and quality of life, the implications of this for workforce retention offered insight into the attrition rate post training of doctors in the UK. A consultant reported that (i6) *“people are leaving the NHS and they are going overseas”*. Five doctors referenced America and Australia specifically as offering an optimal workplace conducive to doctors’ well-being and quality of life compared to the UK; one Junior Doctor (JD) described his comparative experience, (i9) “*I worked in Australia and did those shifts (.) but they weren’t hard because you had so much down time (…) but generally in the NHS you don’t really stop at all*”, he contemplated; (i9) “*I didn’t think it was possible in medicine to have a good work life balance (.) I kind of just accepted it (…) but then when I went to Australia I was like ohh”*.

The 24 doctors represented an equal proportion of men and women (*n* = 12), though FGI-2 was female-dominant (four female, two male). All reported their gender-identity as cisgender. Sex was an influential theme to female doctors, who reflected subthemes of “capacity to attend to sex-specific health behaviors and physical needs”, in addition to the theme of sex differences, which incorporated within-speciality sex-dominance, gender-stereotyping and discrimination; (i2) ^”^*I work in a particularly macho macho environment which means that people are less likely to admit to needing help”*. Though not all the doctors expressed these concerns, this female consultant cardiologist identified the NHS as a culture rife with “toxic masculinity”: (i2) *“our on-shift health behaviors are influenced by toxic masculinity ‘cause I work in a very male dominated field”.*

Family status was a key vulnerability predictor in both sexes, with subthemes of dependent commitments and subsequent impact on shift work, inability to recover off-shift, employment inflexibility, and consequences for loved ones from both acute and cumulative impact of care responsibilities at home. A 42-year old male consultant shared that (i6) *“I know there will be implications and one of them I’ve neglected my family and my friends (…) how long I can do that for in the current climate is difficult to say”*. However, the connection to children, particularly, though physically challenging, was often described as a protective factor—a psychological buffer to counteract work stress, despite its impact on off-shift recovery potential. A consultant mother with young children described that:

(i2)Only about 10% of the consultants are female (…) so it’s the luck of the draw as to whether or not you’ve got a departmental head who’s prepared to allow you to work flexibly (…) me taking time out to do something that will ultimately be a stress reliever for me (…) my God my kids are really stressful (.) sometimes they don’t sleep (…) but actually it’s nice to be able to give my daughter a bath and have cuddles (.) I don’t get to do that most of the time

Doctors’ ages ranged from 23 to 64 (median 34.5) years. The IDI cohort’s age range was 24 to 64 (median 34.5). In FGI-1, the age range was 34 to 64 (median 45) and in FGI-2 was 23 to 63 (median 29). Age was discussed with two dominant themes: physical capability (particularly the capacity to cope with neglect of health needs), and maturity of perspective.

Occupational status was deemed the most influential modifying EHBM variable. The 24 recruited participants were employed full-time by the NHS at 13 different hospitals across the UK (England *n* = 20, Scotland *n* = 1, Wales *n* = 3). Fifteen (62.5%) were JDs, and nine were consultants (37.5%); the distribution of these per mode of interview was *n* = 9 (64.29%) JDs and *n* = 5 (35.71%) consultants in the IDIs, FGI-1 had equal (50%, *n* = 2) representation of each, and FGI-2 included *n* = 4 (66.67%) JD’s and *n* = 2 (33.33%) consultants. Accordingly, nine participants (37.5%) had qualified as doctors within three years of their interview, six (25%) between 2010 and 2019, three (12.5%) between 2000 and 2009, two (8.33%) between 1990 and 1999, and four (16.67%) had qualified between 1980 and 1989.

Occupational seniority influenced on-shift health behaviour and beliefs. A 27-year-old JD admitted that when feeling that his health was impacting on competence; (i11) *“depending on who your seniors are it can be quite difficult to kind of voice at times”*. The doctors described that professional status and occupational responsibilities predict self-efficacy and create health behavior disparities. These disparities related to identity: (i3) *“the FY1’s (foundation-year-one’s) there’s this drive to constantly be doing something to seem productive and they don’t take a moment to step aside and think (.) gosh they should probably just have a glass of water and something to eat”*, and also to professional responsibilities: (f2.2) “*for the juniors you know it’s the intensiveness of that 12–15 hour period (…) for the consultants it’s not so intense because they’re not physically on the ward*”.

### Modifying psychological characteristics

Doctors identified predisposing psychological characteristics including personality, bi-directional trait effects associated with high self-expectations, and mental health. They described adaptive and maladaptive cognitive-behavioural reactions to the doctors’ on-shift health needs. The sub-theme of maladaptive perfectionism dominated as a mechanism for further themes of vulnerability to burnout. Doctors described psychosocial pressure and social vulnerability as catalysts for this, a consultant shared that: (i2) ^”^*there’s a big streak of perfectionism (.) that’s part of our phenotype and that can mean doctors are less likely to stop and take a break until they’ve got it right”*. A senior consultant described that (f2.2) *“the perfectionism (.) when students become JDs and suddenly there’s this huge pressure (…) what was an asset becomes to some extent to a drain”*. This was a recurrent reflective focus, particularly in FGI-2:

(f2.4)Doctors who push themselves to the limit are are seen as being the model

(f2.2)They always used to in the past

(f2.1)Yeah yeah

(f2.4)Yes I mean there is still that kind of stigma nowadays (.) so if you’re not devoting absolutely everything to work then you may not be seen as the ideal doctor

(f2.2)Right yeah

(Interviewer)Yes

(f2.3)I agree with that and it’s really hard to find the balance between unhealthy perfectionism and just being ambitious and work-driven

Positive bidirectional trait effects associated with high self-expectation included sub-themes of ambition, reflecting further themes of diligence, work ethic, perceived success, pride, and adaptive coping strategies. Typical associated statements with these sub-themes were (i11) “*the reason we do the job is ‘cause we want to help people and make a difference*”, and (i13) “*doctors are very driven and focused individuals who have a strong sense of duty*”. However, bidirectionality was often also situated in the negative elements of self-criticism and perceived failure. An indicative quote from a JD described the impact on health behaviour (i7) “*I often forget to eat (.) I think doctors can be quite obsessional and over-focused on their work*”.

Mental health was another identified theme, with prevalent subthemes of anxiety, depression, psychosocial vulnerability; (f1.3) *“working shifts are so busy (…) that leads to a lot of stress and anxiety (.) a lot of doctors don’t really have time to focus on themselves and what they need before making decisions that can be life or death (.) I think a lot of doctors show up very stressed and they don’t really seem in the best state to be making those decisions”*. Low emotional competence, anxious-avoidant strategies and varied responses to stress and trauma were also described.

#### Further contributing predisposing contextual factors

The doctors identified _[T1]_systemic position and _[T2]_work context as themes predictive of on-shift health-protective behaviour. Theme one described subthemes of the challenges of the political climate and associated policies that impacted their perceived systemic alliance, such as the impact of Brexit; (f2.4) “*with COVID and Brexit a lot of people working in healthcare realized just how understaffed and unfair it really was sometimes working here (.) so a lot of people have left and that’s made us even more understaffed*”. Working conditions, including resource inadequacy, loss of staff, alongside NHS instability with austerity measures impacting salaries and strategic work performance measures were central. Theme two pertained to subthemes of compromised working conditions with high workload and skeleton staffing.

Departmental variances were noted between acute versus chronic wards with different specialities having varied stereotypical approaches to work ethic and morale; (i11) *“some specialties (…) I don’t name and shame generally but they don’t seem to be as welfare oriented because they’re very competitive and intense”* another JD echoed that (i12) “*in different specialties (.) for example in A&E they do look after you well (.) they make sure you do get your breaks and there’s help*”. Personality was also described concerning speciality-identity effects by doctors; for example, (i12) “*each specialty attracts certain people (.) certain personalities*”.

### Perceived susceptibility

The EHBM proposes that the “motivation” for health-protective behavioural action occurs when individuals believe they are “susceptible” to an adverse outcome, that the “severity” of not actioning a health behaviour would negatively impact and that the “benefits” of “action” outweigh the “barriers”. Awareness of subjective perception of vulnerability to health risk varied, some doctors were highly cognizant, reporting (f2.3) “*I suspect that we are one of the worst professions at being healthy on-shift*”. This domain was dominated by two central emergent themes: _[T1]_low autonomy and _[T2]_minimization of vulnerability. A senior doctor reflected that (i10) “*my generation of doctors worked ridiculously long hours when we were JDs and fortunately this is much better now but still doctors become overwhelmed because of the work ethic and this can result in health problems*”. Some staff accepted that they could not attend to either their patients or their own health needs in the way they would wish to on-shift. Doctors often minimized the likelihood of experiencing adverse health outcomes or diminished competency outcomes, others understood the predictive susceptibility, in FGI-2 a JD (f2.4) and a consultant (f2.2) shared their concerns:

(f2.4)There can be very severe consequences because your mental health will suffer (.) your physical health will suffer and ultimately the health of the patient by doing these very self-harming things we suffer as well which is completely counterintuitive

(f2.2)We know in terms of physical health that shift work is associated with a substantially higher cardiovascular morbidity and mortality than those not on shift work (.) we know stress dramatically increases the risk of variety of disorders (.) stress and lack of sleep put your cortisol up so it alters mental function

### Perceived severity

Perceived severity refers to a doctor’s assessment of the magnitude of potential consequences of their health behaviour. If a risk is not deemed severe enough to warrant behavioural action, the EHBM predicts a reduced likelihood of change. Dominant themes of _[T1]_personal impact, _[T2]_work performance, and _[T3]_occupational norms were described in response to their perceived severity of neglecting their on-shift health needs.

(i3)A colleague used to get terrible migraines if she didn’t drink enough water (.) she was totally debilitated (.) she asked the health and safety officer if she could take her water bottle on the ward round with her because she needed to keep hydrating in a hot ward and she was told no (…) she was told it was an infection control risk (.) she explained she would be washing hands and said she’d be wearing PPE seeing patients but again the clinical workload took priority over her health

In theme one (personal impact), subthemes of behavioural gravity (including risk-taking, self-sacrifice and understanding of needs), and consequences (concern from loved ones, cumulative effects, and impact on health). Doctors generally minimized severity despite disclosing life-threatening and life-limiting examples of neglecting their on-shift health needs. Glaring adverse events relating to inaction of attending to needs were typical consequences of health neglect ranging in severity and impact. A consultant described the impact:

(i2)I almost died when I was pregnant with my first child (.) I was 30 weeks pregnant (…) I had severe preeclampsia (.) but I ignored my symptoms because I was feeling guilty because colleagues were covering my work (.) I had a very difficult pregnancy and I was swelling up and my blood pressure was going up (.) and I was really breathless but I carried on (.) I just couldn’t cope and then I got really unwell (…) I remember where I really thought I was unwell was when a patient said to me (.) “Are you OK doctor?” literally three days later I was having an emergency caesarean section after having a seizure (…) I had preeclampsia (.) I just carried on and my colleagues saw it as well (…) one of the consultants initially said in retrospect he thought she really doesn’t look well (.) Well why on Earth didn’t they say? (.) that permission might have been enough for me to say (…) I need a break and that’s had lifelong consequences for my child who’s born premature and myself and it’s a very traumatic start to life (.) he’s got health problems now and that all stemmed from maladaptive coping mechanisms at work

Perceived severity related to both doctors and patient impact. One JD described that (i9) “*towards the end of particularly a 13-hour night shift you always dread like getting an arrest call at six or seven am because you are noticeably slower in terms of your thinking and your energy*”, and he described the risk to life being not confined to his patients:

(i9)I’ve been paying for a hotel after night shifts because I know I won’t be safe to drive because I have lots of colleagues who’ve crashed after night shifts (.) it’s like being under the influence of four or five pints of beer (…) I’ve fallen asleep on the M4 motorway once coming home from a night shift

Theme two (work performance) included risk to patients, attributed to inaccurate risk appraisal and the impact of impaired decision-making. This was a motivating factor for several doctors, with one consultant describing how it impacted their self-regulation and competent practice: (i4) ^“^*every couple of hours I’ll stop and have a drink (.) maybe go to the toilet and go back to it because I become very inefficient and I start getting patients mixed up doing a ward round it all starts to blur into one”*. The third emergent moderating theme of occupational norms incorporated subthemes of psychosocial collusion, minimization and impaired autonomy. There was a consistent thematic acceptance that decisional balance reflected risk management strategies rather than striving for absolute safety in a milieu of systemic threat.

### Perceived benefits

If the benefits of health-protective action outweigh the barriers, then the EHBM predicts reasoned action. Doctors identified common thematic benefits: Theme one was improved work ambience, with subthemes of normative shifts and positive milieu. Theme two was well-being, including quality of life, psychophysiological health benefits and improved cognition; (f2.2) *“a healthier doctor provides better care to their patients because when you’re dehydrated or you haven’t slept enough you tend to be a lot less rational”*. A senior consultant reflected (i10) “*my generation of doctors worked ridiculously long hours when we were junior doctors and fortunately this is much better now (.) but still individuals become overwhelmed because of the work ethic and this can result in health problems*”. Lastly, theme three was fitness to practice, comprising subthemes of improved competence, patient safety, staff retention, shift endurance and increased longevity; a consultant asserted that (i1) ^“^*by compromising their own wellbeing ultimately there is the potential to compromise the care of patients”.*

Doctors reported cognizance of a multitude of positive outcomes and benefits associated with engaging in health-protective behaviours on-shift emphasizing the shared premise that (i8) *“I think my health is very important in my work”*. A JD identified that prioritizing health-protective behaviour on-shift resulted in (i7) “*better physical and psychological health leads to better functioning and a greater ability to focus on the needs of the patients”*. Although, these benefits were typically dismissed against perceived barriers.

### Perceived barriers

The EHBM asserts that behaviour modification transpires when individuals perceive a necessity to alter their existing behaviours and hold the conviction that such behavioural changes will result in favourable outcomes, weighed against a reasonable cost or effort. Perceived barriers were _[T1]_occupational beliefs and perceptions, _[T2]_physical and procedural barriers, and _[T3]_workload. These barriers function as cues to (in)action despite an identified biological prompt (e.g., exhaustion, hunger) were lack of staffing to cover, not wanting to be seen slacking.

Theme one within this domain reflected the collective norms, expected demands and systemic groupthink in the medical culture that prioritizes work and rewards selflessness. Adverse consequences associated with deviating from _[ST1a]_collective norms and _[ST1b]_systemic groupthink were considered psychosocially and occupationally detrimental in a professional milieu that rewards selflessness; a consultant disclosed that (i10) “*although the workload is a major barrier (…) the major barrier is the mindset that is common in doctors (…) which almost encourages one to neglect one’s own health”*. Behavioral activation was highly predicted by the doctor’s self-efficacy, which predicted their orientation to cues to action.

Doctors’ perceptions of _[T2]_procedural barriers and obstacles were often at a professional identity level in addition to being systemically functional. Mediating factors included the perceived magnitude of obstacles to health-protective behaviour. As a representative quote from a JD highlighted:

(i3)You can’t use the patient’s cups in Scotland we’ve banned disposable plastic now (…) so you can’t even have disposable like a water cooler with disposable cups for staff (.) even if there was it would have to be in the break room and again that’s interruption to workflow (…) when you work in an area where that’s not the case such as in in my teaching role you do notice a huge difference (.) just being able to get a drink of water is everything

Night shift was described as a strong predictor of dietary behaviour, with consistent reports that (f2.2) “*on night duties often the restaurants are closed so there’s a vending machine but the food is not very healthy (.) so even if you find the time to do it (…) I think you tend not to eat very well*”. The theme of workload was dominated by a perceived lack of time, with doctors typically describing; (i9) “*there wasn’t any time for lunch because we just carried on all day*”.

#### Further psycho-socio-systemic mediators

Inductive emergent analysis identified two overarching themes as additional mediators and motivators through open inductive coding. The primary theme, _[T1]_“health service operational culture”, comprised subthemes of a critical work ethos related to the culture that promotes negative peer pressure among employees; (i9) “*the system sort of breeds it (.) people resent people when they’re taking a day off sick (.) because it affects them and they have to do more work*”. All described socio-systemic burnout risk behaviours as a prized state within NHS organizational culture; (f2.3) “*we tend to prioritize our patient’s health which inadvertently makes us deprioritize our own health*”. Within the primary theme, further sub-themes of professional and personal dissonance, permeated role expectancies, disenchantment, and reporting no faith in policy rationale often resulted in non-compliance with regulations.

IDIs elicited different responses to FGIs and were notably shorter in response time-per-participant. Differences in group dynamics between the IDIs and FGIs was marked. Analyzed within a more interpretive analytic epistemology, latent underpinning mechanisms were apparent that may have been attributable to their being within staff team (i.e., colleagues) at varied career stages, which may have stifled open communication but did expose the dynamics likely to contribute to the dissonance and de-prioritization of self-care as seniors repeatedly dismissed or minimized JDs experiences. In FGI-2 with an acute medical team working in central London, this was particularly apparent in a dialogue between two consultants—a 47-year-old female (f2.6) and a 63-year-old male (f2.2), and a female 25-year-old JD (f2.4):

(f2.6)And in some ways the culture is a little bit better than it used to be (.) I think the cultural issue has improved slightly

(interviewer)Yes?

(f2.2)In that you know in years gone by junior doctors used to work absolutely horrendous hours and that was sort of built into the whole mindset that (…) their hours have improved a little and that’s impacted on the culture but I still think it’s far from ideal

(f2.4)There’s still a lot of work for junior doctors to do and it can be incredibly stressful and (…) you just have [Interrupted]

(f2.2)Yes and I think the other issue (.) and it’s probably relevant to this is that you know (.) we’re working as an acute team but there are many parts of medicine which no longer have teams because it’s based on shift work (…) they’re actually working really quite alone in isolation doing a particular shift and not feeling part of a team (.) and the team is really quite important for supporting each other

(f2.4)Yes I agree

The secondary semantic theme of “Governance” reinforced this further, this was characterized by _[ST2a]_disconnected understanding, which reflected regulatory priorities that are disengaged from policy outcome. The frustration at strategic disconnect and operational policies that were barriers to doctors’ health needs were characteristic according to one consultant:

(i6)the bureaucracy is so huge and the managers (…) they don’t listen and that’s why NHS is being privatized and outsourced (…) I know of several organizations who are making their making good bucks out of NHS is just because NHS is being run by poor managers (…) on paper managers would say everybody needs a break (…) but in reality that doesn’t happen

Doctors described tokenistic and ineffective messaging about health promotion, including from the British Medical Association (BMA) and General Medical Council (GMC) (f2.2) “*the GMC make a big issue of this but it’s still part of the mindset and the culture of medicine (…) is perhaps to ignore one’s own health (…) you know that’s historically been part of the culture*”. These misaligned governance objectives and policy implementation strategies reflected a disconnect from frontline needs where systems need to support health.

### Self-efficacy

**Figure 2.**
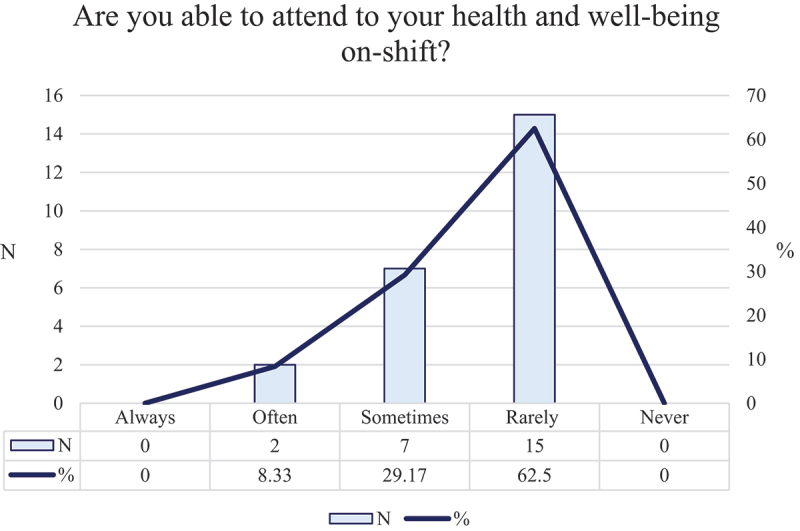
Doctors perceived ability to attend to health and well-being needs.

Self-efficacy refers to a doctor’s belief in their ability to successfully perform health-protective behaviours through their capacity to overcome barriers and their perceived ability to initiate and sustain these health-related actions. If self-efficacy supports these motivating factors, the model predicts connection with cues to health-protective behaviour, and resultant action. In the survey, doctors were asked, “*Are you able to attend to your health and well-being on-shift*?” Fifteen doctors (62.5%) indicated a “rarely” response from the 5-point options presented in Figure 2.

Self-efficacy was a dominant theme as a proximal factor of decisional reasoning and behavioural action. Diminished self-efficacy was attributed to themes of; workload-related time constraints, staffing, the reactive milieu associated with patient needs, emergencies, insurmountable obstacles (e.g., night-shift food availability), PPE and infection control measures. Colleague judgement was reported if doctors took breaks, one describing the impact being; (i9) “*from day one as a junior doctor you kind of realize you have to work all the time and you have to not be sick and not take holidays (.) and you feel guilty if you are sick or you do take a holiday ‘cause you know it’s going to double everyone’s workload*”. These perceived pressures adversely impacted self-efficacy.

Limited perceived control was normative, exceptions were typically attributed to seniority or a disregard for policies that did not make sense to them. One doctor justified his stance of prioritizing health over infection control policies because (i9) “*in the NHS particularly there are a lot of stupid rules*.” Consultants who were less ward-based and were not as concerned about judgement from junior colleagues had enhanced self-preservation beliefs, greater self-efficacy and autonomy.

### Cues to action

The diverse range of cues that may prompt doctors to take action included _[T1]_extrinsic prompts and _[T2]_intrinsic cues. Extrinsic prompts included subthemes of _[ST1a]_operational staff prompts, including top-down modelling, culture shift, health education, senior doctors monitoring staff needs, formal cues, and systems to cover patient caseload, alongside _[ST1b]_strategic stimulus, incorporating clinical guiding principles, systemic expectations, policy dissemination and monitoring audits. One doctor mentioned, (i5) “*when you’re in such a very high pressure and busy workplace you often forget a lot of things (.) so having that reminder alongside (.) it can prompt you to drink and to remember that your needs are just as important to your health and for your patients*”. An example of how they implemented this in top-down modelling was:

(i3)I’ve often spoken to FY1’s in their first roles and it will get to two o’clock and I’ll say (.) have you had a break yet? Have you sat down and had something to eat or drink some water (…) and they’ll say no I was busy doing discharge letters so I’ve not gone to get a glass of water and I just think (…) you’ve got to look after yourself a bit better

Intrinsic cues were perceptually reliant on self-awareness; understanding of psychophysiological impact, and cognizance of diminished well-being influencing patient care, _[ST2b]_activating and provoking factors of self-motivation, personal responsibility-taking and anticipatory regret. _[ST2c]_Cognitive-behavioural cues included planned behavioural intent, self-directed prompts and homoeostatic reactivity. A JD identified that (i7) “*a really good cue for me to know when to take a break is being aware of my own needs (.) although that’s quite difficult sometimes when it’s very busy*”. Doctors employed diverse adaptive and maladaptive cognitive-behavioural strategies to respond to their needs. One doctor articulated the decisional reasoning balancing this appraisal of clinical guiding principles and personal health needs:

(f1.1)The demands are huge but there’s also a degree of choice (…) part of it is about the personality issues (.) in some ways I think we rationalize that as putting patients first which of course we should but harming ourselves harms our patients too

### Action

Various inductive themes emerged as central to the doctors’ experiences of actioning and sustaining the implementation of behaviour. These themes primarily concerned their beliefs about which specific behaviours to prioritize, what was realistically actionable within the context, and any motivating factors.

#### Doctor-identified priority on-shift health behavior

**Figure 3.**
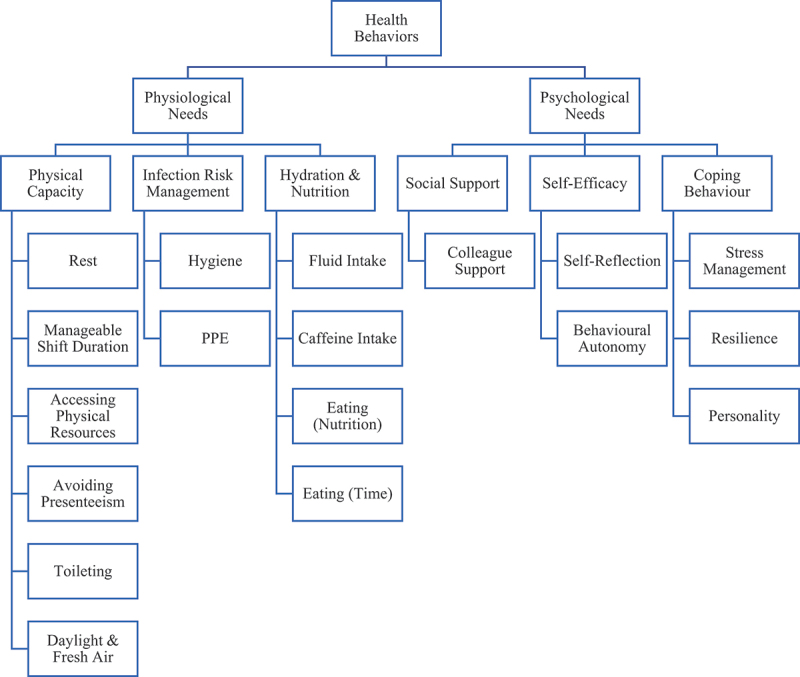
Doctor-identified on-shift health-protective behaviors.

In an open-text survey question, doctors were asked to “list as many on-shift health-protective behaviors as they could think of”. This analytical frame was used to code doctor-identified priority behaviours within the interviews (Figure 3).

Alongside pre-shift sleep, eating and drinking were deemed the priority health behaviours that would most significantly impact well-being and subsequent patient care. A representative quote included, (i5) “*I don’t think that I drink as much as I should*”. Many acknowledged the impact on their competence (i7) “*I don’t perform as well unless I make sure I’m adequately hydrated (.) eating I forget to do*”. Behaviours were predominantly explicit; one doctor reported typical concerns regarding caffeine intake, (i9) “*I’m now pretty much caffeine dependent*”. Eating was subdivided for the distinct contextual relevance for some as having time to simply eat. For 12 doctors, the hospital workplace was a factor regarding the nutritional content of on-shift eating behaviour, (i9) “*in hospital there’s chocolates everywhere (.) patients bring stuff in so there’s always stuff on the ward*”. Whereas exercize and fitness-related behaviours were described as manageable or enhanced by their work; (i9) “*I got a step counter actually (…) on my 13 hour shift I did 14,000 steps which is loads considering it was just walking within a hospital*”.

#### Action-focused mechanisms of change

Doctors deployed various strategies to balance patient needs, colleagues’ expectations and their health. Adaptive mechanisms of decisional reasoning were explored concerning mediating risk trade-offs for competence and patient safety. The EHBM-influenced interview schedule offered prompts and probes that elicited solution-focused proposals for a range of doctor-informed interventions: (i11) ^“^*big things would be to address the availability of stuff (.) so the physical hierarchy of needs (.) availability of immediate water (.) food you need is the big thing and then I think just general culture”.*

Doctors shared positive experiences of actioning health-protective behaviour; (i11) “*once or twice a week someone will come round on A&E with a drinks trolley with biscuits and be like you need to drink (.) which is really nice and it’s often led by one of the senior registrars*”. In FGI-2, the potential for protective prompts was demonstrated: (f2.5) “*If we try to get across that it’s a team responsibility to look out for each other and to prompt each other to take breaks to drink*”. Senior colleague endorsement or a strong sense of self-efficacy (associated with seniority) were deemed the strongest predictors of action; (i1)*As medical students (.) but also as JDs there needs to be some formalized system to state to them it’s not just good for you to do these things it’s the right thing to do for you and your patients.*

Further practical and resource-based actions were identified; (i12) “*I think culture is very difficult to change (.) it takes a while (…) but do the practical things that you can do (.) making sure people have easy access to water, food and resting spaces*”. Another echoed (f1.4) “*it would be good if within each ward there could be more nutritious places that we could actually get drinks and maybe snacks*”. One doctor indicated this provision may have broader work-satisfaction benefits (i12) “*with strike and pay and doctors not being paid enough I think that probably a lot of people echoed the same that if better rest facilities were provided people would be happier where they work*”. A JD identified a beneficial self-directed prompt: (i8) “*setting reminders is a way to help myself on the phone (…) I often will set alarms for my well-being*”, doctors also described dietary monitoring apps to aid self-regulation without reliance on physical cues. Another doctor reinforced this self-regulation by acutely identifying that (f2.3) “*we have to look after ourselves enough to consider the patient’s needs*”, which epitomized the overarching thematic aspiration within the “action” domain.

## Discussion

Doctors with diverse career stages and demographic characteristics reported a plethora of subjective experiences, yet when mapped to the analysis framework, there were strong thematic consistencies in modifying psychological characteristics and health-motivating beliefs in the domains of the perceived barriers, susceptibility, severity, benefits, and cues to action. Key thematic findings were the mechanisms of dissonance embedded in their identity as “doctor” and how the socio-systemic culture fosters incongruous beliefs that operate as barriers to health-protective behaviour. The mediating impact of self-efficacy underpinned all interviews as a bidirectional health-protective and self-detrimental factor. Personal caregiver responsibilities, occupational role (including seniority) and occupational context influenced self-efficacy in engagement with health protective behaviours. Although personal caregiver responsibilities were not captured in the contextual survey, they were a key inductive theme in the thematic analysis; therefore, its inclusion in any future demographic surveys of healthcare professionals is recommended. Seniors minimized the adverse health impact on juniors. Recognizing this context provides clues to informing interventions and policies to promote a safer workplace milieu, which has the potential to positively affect behaviour by enhancing perceived and concrete permissive self-efficacy.

Doctors in this study were cognizant of their diminished health behaviour’s counterintuitive and detrimental effects on their well-being and competence. The volitional components of this dissonance poses challenges for cognitive appraisal-based health-promotion interventions were attributed by them to perceived psycho-socio-systemic organizational culture pressures that prize self-sacrifice, these are embedded as a consistent and persistent health-detrimental research finding in this occupational culture worldwide Fältholm (2007); McCain et al. (2017). As the doctors in this study highlighted, it is also essential to consider how these findings relate to and impact the diverse health service workforce in the NHS and across global health systems. The doctors in the interviews highlighted the broader healthcare workforce effects, particularly of COVID and Brexit, and comparable challenges were faced in navigating policy-based barriers to health-protective behaviour at work within this occupational context.

The findings elucidating the ramifications of COVID-19 on doctors aligned with concurrent research investigations, particularly regarding moral distress and lack of organizational support (Brune et al., 2024; Cubitt et al., 2021). Nevertheless, utilizing the EHBM facilitated a more comprehensive exploration focused on the process aspects related explicitly to on-shift health-protective behaviour impact. The EHBM has been utilized to predict preventative health behaviours (Shitu et al., 2022) and design behavioural prevention programs (Jalilian et al., 2014; Shahnazi et al., 2019). Adaption was necessary to adequately account for the psycho-socio-systemic context influencing health beliefs and behaviours; these domains needed to be explicitly factored into the original theoretical framework. Nonetheless, the model offered a worthwhile exploration tool for beliefs and influences associated with mechanisms of action and motivations for augmenting health-promotive behavioural change. Qualitative inquiry within the model enhanced the depth of understanding, enabling exploration of the subtleties, nuances, and cultural factors that shaped health-related decisional reasoning.

Thematic analysis has recurrently been criticized for not adequately addressing power dynamics; therefore, the positionality of the researchers was declared. Efforts were made in this study to enhance reflexivity, transparency, rigour, and the applicability of findings. The mixed methods and combined deductive and inductive analytic approach drove a comprehensive interpretative process less reliant on explicitly analyst-driven epistemological biases. The survey enriched findings by integrating contextual quantitative data, providing a more comprehensive understanding. Enabling doctors to identify their on-shift health protective behaviours in an open survey question used for coding the transcripts was effective; it provided an occupationally apposite approach that would not have been possible with a generic health behaviour checklist.

Psychosystemic mediators were a key inductive theme in this study, this has both operational and systemic implications. The GMC has recently outlined the expected behavioural standards of patient care in their “*Good Medical Practice*” guidelines (General Medical Council, 2024). These professional standards guidelines that came into effect in January 2024 outline the inclusion of the prioritization of patient needs, standards of competent care, respect for colleagues, integrity, honesty and health promotion for patients and the public. These values, principles of care and standards include comprehensive considerations regarding competent practice and patient care. However, they do not address the health needs of the workforce or the implications of their well-being on practice and subsequent patient care. In contrast, examining broader global approaches, The World Medical Association’s “*Declaration of Geneva*” includes “I will attend to my own health, well-being, and abilities in order to provide care of the highest standard” (World Medical Association, 2024, p. 1). The GMC practice guidelines may potentially be extended to include the practice issues raised in this study. However, the doctors in this study described low regard for what they described as the current misaligned and disconnected strategies by both the GMC and BMA in the health promotion of medical staff, so there may be further barriers to overcome before realignment and compliance are consistent with strategic policy.

This study reflects the experiences of 24 doctors from hospitals in the UK at varied career stages, specialities, demographics, and geographical locations. This number was sufficient to identify and explore a range of diverse insights and beliefs. This study did not seek to furnish statistical inferences or findings generalizable to broader populations, but enabled a latticework to understand how norms, social dynamics, and systemic factors may shape beliefs and behaviour. Future recommendations include harnessing the rich interviews to gain insight and solution-focused opportunities for prospective health promotion interventions may be formulated in alignment with the EHBM, incorporating tailored messaging that explicitly addresses health-motivation domains, specifically targeting doctors’ beliefs and cues to action, thereby enhancing their efficacy through contextual relevance, and may also inform future medical education.

This study is the first to apply the EHBM to understand doctors’ on-shift health behaviour and its effects during unprecedented systemic pressures on staff. It enabled key doctor-informed recommendations from this research on individual and broader systemic levels. The inclusion of both IDIs and FGIs in this study further emphasized occupational disparities and demonstrated the emergent psychosociosystemic top-down influences on health behaviour. Applying the EHBM framework, this study discerned the determinants of the doctors’ foundational beliefs and elucidated the decisional reasoning and mechanisms of behavioural action that influenced their health. These salient beliefs constitute crucial factors in modifying behaviour and may tentatively function as targeted objectives for interventions to foster health-protective measures as the thematic mapping of the EHBM offers a framework to examine these risk trade-offs as mediators that may be leveraged. Since its inception, HBM has been applied as an effective model to develop behaviour change interventions, and it has been found to motivate action through its process orientation towards solution-focused action (C. J. Jones et al., 2014; C. L. Jones et al., 2015; Thomas et al., 2024). Although the aim of this study pertained to exploratory understanding, an unanticipated outcome of the HBM-informed interview schedule in this study was that during these semi-structured interviews, the majority of doctors reflected on their health-detrimental behaviours and identified possible prospective actions and cues to actions to address these. Therefore, the inductive analysis captured action-oriented responses a posteriori that had noteworthy action-oriented participatory effects.

The perspectives of the doctors in this study particularly align with Gerada’s (2020) insights regarding the perfectionistic mindset of doctors creating well-being barriers, in addition to many of the recommendations by Gerada highlighting the impact of doctor well-being on patient care and appeal for greater understanding and support of needs (Gerada, 2019). As the doctors in this study identified, improving workplace well-being within the health service necessitates a multi-level approach to address personal, operational, and systemic challenges. The policy and practice recommendations informed by the doctors in this study were as follows. On a personal practice level, many intrinsic cues were described as perceptually reliant upon self-awareness, reflective practice, and self-efficacy; therefore, occupationally apposite health education promoting awareness of the effects of diminished well-being influencing patient care while encouraging self-efficacy may activate greater health motivation. On an operational level, doctors identified diverse cues that may prompt staff to take health-protective behavioural action, including extrinsic staff prompts, top-down modelling, monitoring staff needs, and formal cues with support to cover patient caseload. Finally, systemic and strategic level reviews of clinical guiding principles, resources, expectations, policy dissemination, cultural shifts, and monitoring audits to identify implementation barriers may support the gap between policy and practices in staff well-being. Alongside pre-shift sleep, eating and drinking were deemed the priority health behaviours that would most significantly impact well-being and subsequent patient care; ensuring the accessibility of nutritious food and drink on shift is a fundamental requirement for all healthcare staff. By addressing doctors and broader healthcare staff’s well-being from this multi-level perspective, there is potential to encourage a more sustainable workforce and foster a climate conducive to improved patient care.

## Supplementary Material

Supplementary File 2 Comprehensive Thematic Mapping.docx

Supplementary File 1 Research Protocol.docx

## Data Availability

Supplementary files associated with this study are provided. Further data that support the findings of this study are available from the corresponding author upon reasonable request.

## List of Abbreviations

A&E: Accident & Emergency
AR: Anonymized
BMA: British Medical Association
COVID-19: Coronavirus Disease
EHBM: EHBM
(f): Focus Group Participant
FGI: Focus Group Interview
[FT]: Further Theme
FY: Foundation Year
GMC: General Medical Association
HBM: Health Belief Model
(i): Interview Participant
ICU: Intensive Care Unit
IDI: Individual Depth Interview
JD: Junior Doctor
M4: Motorway 4
NHS: National Health Service
OS: Online Survey Software
[ST]: Subtheme
[T]: Theme
UK: United Kingdom
ICU: Intensive Care Unit
A&E: Accident and Emergency
