# Supplementary material for: “Are you OK doctor?” An expanded health belief model exploration of doctors’ experiences and perspectives of on-shift health behaviour
Source: Int J Qual Stud Health Well-being. 2024 Aug 5;19(1):2388795. doi: 10.1080/17482631.2024.2388795 (PMC11305022; doi:10.1080/17482631.2024.2388795)
Supplement: Supplementary File 2 Comprehensive Thematic Mapping.docx [file ZQHW_A_2388795_SM2515.docx]

**Supplementary File 2.**

*Comprehensive Thematic Mapping*

***Theory Derived Code [TDC]***

**The Theory Derived Coding of the** EHBM **associated interview discussion was analyzed using a combination of deductive and inductive coding methods.** Broad quotations were further extracted and deductively coded for each theoretical domain, and inductive candidate coding was undertaken on additional factors and themes.

**Table 1.**

*Factor mapping of extracted data*

| **Data extracted for thematic analysis** | | |  | **Factor Mapping of Extracted Data** | | | | | | | | | | |
| --- | --- | --- | --- | --- | --- | --- | --- | --- | --- | --- | --- | --- | --- | --- |
|  |  |  |  | ***Predisposing Factors*** | | | ***Health Motivation*** | | | | | ***Behavioural Activation*** | | |
| A | B | C |  | E | F | G | H | I | J | K | L | M | N | O |
| Timestamp | Speaker | Extracted Pertinent Discourse |  | Demographic Variables | Psychological Factors | Further Contributing Factors | Perceived Susceptibility | Perceived Severity | Perceived Benefits | Perceived Barriers | Additional Mediators | Self-efficacy | Cues to action | Action |

**Research Objectives Derived Code [RODC].** Interview transcripts were inductively coded for a data driven-exploration of: Systemic experiences related to the NHS (CL-NHS); experiences of the contemporary landscape including COVID-19 (CL-CVD); health behaviour (HB)**;** action research (AR); and competence (C)

**Data Derived Code - [DDC-prominence and keyness]. Inductive data derived coding was also undertaken throughout to identify any themes that were not apriori specified, particularly relating to occupational context which was coded for prominence and keyness (OC- coded for prominence).**

**Domain-mapping of extracts.** To address the study objectives, thematic mapping was operated as an iterative recursive framework for organization and interpretation.

**Listed themes and subthemes within the Health Belief Model domains**

*The Expanded Health Belief Model (EHBM) is a psychological framework that explains and predicts health behaviors by considering and encompassing six key domains: perceived susceptibility, perceived severity, perceived benefits, perceived barriers, cues to action, and self-efficacy. These domains collectively influence individuals' perceptions, attitudes, and behaviors related to health promotion and disease prevention.*

**Domain: Demographic Variables**

Theme 1. Age

Subtheme 1a. Physical capability

Further theme. Capacity to cope with neglect of health needs

Subtheme 1b. Maturity of perspective

Further theme. Expectations

Further theme. Resilience

Further theme. Difference in perspective

Theme 2. Sex

Subtheme 2a. Capacity

Further theme. Sex-specific health behaviours

Further theme. Sex-specific physical needs

Subtheme 2b. Sex differences

Further theme. Within-specialty sex dominance

Further theme. Gender stereotyping and discrimination

Theme 3. Ethnicity

Subtheme 3a. Identity

Further theme. Belonging

Further theme. Values and beliefs

Subtheme 3b. Integration and inclusion

Further theme. Country of working residence

Further theme. Cultural norms and expectations

Theme 4. Occupational status

Subtheme 4a. Professional status and occupational responsibilities

Further theme. Job description determines self-efficacy and creates health behaviour disparities

Further theme. Specialism predicts the demands competing with personal health

Theme 5. Family Status

Subtheme 5a. Dependent commitments

Further theme. Impact of dependents on shift work

Further theme. Employment inflexibility

Further theme. Carer responsibilities

Subtheme 5b. Off-shift recovery

Further theme. Ability to rest off-shift

Further theme. Pressure

Further theme. Support

**Domain: Psychological Factors**

Theme 1. Personality

Subtheme 1a. Maladaptive perfectionism

Further theme. Vulnerability to burnout

Subtheme 1b. Social vulnerability

Further theme. Psychosocial pressure

Further theme. Imposter syndrome

Theme 2. Bi-directional trait effects associated with high self-expectation

Subtheme 2a. Ambition

Further theme. Diligence and work ethic

Further theme. Perceived success and pride

Further theme. Adaptive strategies to cope

Subtheme 2b. Negative trait effects

Further theme. Self-critical and perceived failure

Theme 3. Mental Health

Subtheme 3a. Anxiety and Depression

Further theme. Vulnerability to burnout

Subtheme 3b. Emotional competence

Further theme. Anxious avoidant

Further theme. Responses to stress and trauma

**Domain: Further Contributing Predisposing Contextual Factors**

Theme 1. Systemic Position

Subtheme 1a. Unexpected challenges

Further theme. Political climate

Further theme. Viral risk to population

Further theme. Government policies

Subtheme 1b. Unstable organization

Further theme. NHS funding distribution

Further theme. Appraisal and 360-degree feedback

Theme 2. Work Context

Subtheme 2a. Departmental variances

Further theme. Acute versus chronic wards

Further theme. Within versus without team working

Further theme. Perceived systemic alliance

Subtheme 2b. Compromised working conditions

Further theme. Staffing

Further theme. Adequacy of resources

Further theme. The work is never 'done'

**Domain: Health Motivation – Perceived Susceptibility**

*This domain refers to an individual's subjective perception of risk or vulnerability to a particular health condition or disease. It involves assessing the individual's beliefs about the likelihood of experiencing adverse health outcomes if they engage or do not engage in certain behaviors.*

Theme 1. Low autonomy

Subtheme 1a. Professional Responsibility

Further theme. Acceptance of expected self-sacrifices

Further theme. Letting team down

Further theme. Unhealthy senior role models

Subtheme 1b. Disempowering pressure

Further theme. Fear of non-conformity

Further theme. Unable to abandon the ward

Theme 2. Minimization of vulnerability

Subtheme 2a. Reprioritization

                       Further theme. The patient's ill health supersedes personal health

Subtheme 2b. Needs suppression

Further theme. Age is viewed as a buffer to vulnerability

Further theme. Complacency

Further theme. Health needs as a perceived weakness

Further theme. Denial of symptoms

Further theme. Physical and emotional dissociation

**Domain: Health Motivation – Perceived Severity**

*Perceived severity refers to an individual's assessment of the seriousness or magnitude of the potential consequences associated with a health condition or disease. It involves evaluating the perceived impact of the health condition on one's physical, emotional, and social well-being.*

Theme 1. Personal Impact

Subtheme 1a. Behavioural gravity

Further theme. Risk-taking and self-sacrifice

Further theme. Self-criticism

Further theme. Underestimation of needs

Subtheme 1b. Consequences

Further theme. Concern from loved ones

Further theme. Cumulative impact

Further theme. Impact on health

Theme 2. Work Performance

Subtheme 2a. Risk to patients

Further theme. Inaccurate risk appraisal

Further theme. Impact of impaired decision-making on patient care

Subtheme 2b. Risk to professional integrity

Further theme. Avoiding decision-making when health is compromised

Further theme. Being present is more important than being effective

Further theme. Guilt from medical judgment errors

Theme 3. Occupational norms

Subtheme 3a. Psychosocial collusion

Further theme. Senior doctors minimize the impact on junior doctors

Further theme. Lack of support

Further theme. Ethical judgement

Subtheme 3b. Autonomy

Further theme. Permission to attend to needs

**Domain: Health Motivation – Perceived Benefits**

*This domain involves an individual's evaluation of the perceived effectiveness or advantages of adopting specific health behaviors to reduce the risk of a health condition or disease. It includes assessing the perceived positive outcomes or benefits associated with engaging in preventive or protective health actions.*

Theme 1. Fitness to Practice

Subtheme 2a. Competence

Further theme. Decision making, performance and patient safety

Subtheme 2b. Staff retention

Further theme. Shift endurance

Further theme. Longevity

Theme 2. Improved Work Ambiance

Subtheme 1a. Normative shifts

Further theme. Healthier standards

Further theme. Reduced professional dissonance

Subtheme 1b. Positive milieu

Further theme. Modelling positive health behaviour

Further theme. Staff safety

Further theme. Consideration of others' needs

Theme 3. Well-being

Subtheme 3a. Physical health benefits

Further theme. Lifestyle

Further theme. Reduced personal health risk

Subtheme 3b. Psychological health benefits

Further theme. Buffer to stress

Further theme. Improved Cognition

**Domain: Health Motivation – Perceived Barriers**

*Perceived barriers refer to the individual's perception of the obstacles, challenges, or adverse consequences that may hinder or prevent them from adopting or maintaining certain health behaviors. This domain involves evaluating the perceived costs, effort, or potential adverse effects of engaging in health-promoting actions.*

Theme 1. Occupational beliefs and perceptions

Subtheme 1a. Collective norms

Further theme. Unrealistic expectations

Further theme. Expected demands

Subtheme 1b. Systemic groupthink

Further theme. Systemic culture rewards selflessness

Further theme. Work commitments take priority

Theme 2. Physical and procedural barriers

Subtheme 2a. COVID-19 related protocols

                      Further theme. PPE

Subtheme 2b. Understaffing

Further theme. No staff to enable relief cover

Subtheme 2c. Health resource accessibility

Further theme. Access to food and drink

Further theme. NHS regulations and policies

Theme 3. Workload

Subtheme 3a. Principled obligations

Further theme. Demands on the ward

Further theme. Ethical responsibility

Subtheme 3b. Time pressure

Further theme. Time-sensitive tests and treatment

Further theme. Time stress

**Domain: Further Psycho-socio-systemic Mediators**

Theme 1. Health service operational culture

Subtheme 1a. Critical work ethos

Further theme. Judgement from colleagues

Subtheme 1b. Permeated role expectancies

Further theme. Professional and personal dissonance

Further theme. Appraisal of the payoff of health sacrifice

Further theme. Fear of senior doctors

Subtheme 1c. Disenchantment

Further theme. Non-compliance to policies and regulations

Further theme. No faith in systemic policy rationale

Theme 2. Governance

Subtheme 2a. Disconnected understanding

                       Further theme. Regulatory priorities

Further theme. Disengaged from policy outcome

Further theme. Tokenistic and ineffective attempts at health promotion

Subtheme 2b. Misaligned strategic objectives

Further theme. Policy implementation strategy

Further theme. Disconnect from the needs of frontline staff

Further theme. Systems do not support staff health

**Domain: Behavioural Activation - Self-efficacy**

*Self-efficacy refers to an individual's belief in their ability to successfully perform specific health behaviors required to achieve desired health outcomes. It involves assessing one's confidence in their capacity to overcome barriers and challenges and their perceived ability to initiate and sustain health-related actions.*

Theme 1. Diminished self-efficacy

Subtheme 1a. Time constraints

           Further theme. Workload

           Further theme. Absence of relief medic

Subtheme 1b. Reactive milieu

Further theme. Patient emergencies

          Further theme. Ward staffing requirements

Further theme. Stress and burnout

Subtheme 1c. Insurmountable obstacles to health-protective behaviour

            Further theme. PPE removal risks infection

Further theme. Fear of judgement

Further theme. Food and drink are unavailable

Further theme. Night shift

Theme 2. Enhanced self-efficacy

Subtheme 2a. Self-assured senior authority

Further theme. Increased time off the ward

            Further theme. Self-management of time

Further theme. Less concerned about visible performance

Subtheme 2b. Confidence in health-needs prioritization

Further theme. Self-preservation

Further theme. Autonomy

**Domain: Behavioural Activation - Cues to action**

*Cues to action are external or internal stimuli that prompt or trigger individuals to engage in health behaviors. These cues can include informational cues (e.g., health education campaigns), environmental cues (e.g., availability of resources), or personal cues (e.g., symptoms or feedback from healthcare providers) that motivate individuals to take action to protect or improve their health.*

Theme 1. Extrinsic prompts

Subtheme 1a. Operational staff prompts

Further theme. Top-down modelling

Further theme. Culture shift

Further theme. Health education

Further theme. Senior doctors monitoring staff needs

Further theme. Formal cues

Further theme. Systems to cover patient caseload

Subtheme 1b. Strategic stimulus

Further theme. Clinical guiding principles

Further theme. Systemic expectations

Further theme. Policy dissemination

Further theme. Monitoring audits

Theme 2. Intrinsic cues

Subtheme 2a. Perceptual

Further theme. Self-awareness

Further theme. Understanding of psychophysiological impact

Further theme. Cognizance of diminished well-being in patient care

Subtheme 2b. Activating and provoking

Further theme. Self-motivation

Further theme. Personal responsibility-taking

Further theme. Anticipatory regret

Subtheme 2c. Cognitive-behavioural

Further theme. Planned behavioural intent

Further theme. Self-directed prompts

Further theme. Reactive homeostatic behaviour

**Domain: Behavioural Activation - Action**

Theme 1. Individual activation

Subtheme 1a. Proactive personal initiation

Further theme. Planned behavioural action

Subtheme 1b. Reactive personal activation

Further theme. Behavioural reaction to needs

Further theme. Actions responsive to individual health and work context applied

Subtheme 1c. Self-advocacy

                        Further theme. Deviation from maladaptive social norms

Theme 2. Adherence to systemic policies and procedures

Subtheme 2a. System-wide directives

Further theme. Monitoring and reporting

Further theme. Explicit implemented support

Further theme. Regulatory implementation

Subtheme 2b. Operational policy execution

Further theme. Top-down prompts

Further theme. Visual health promotion prompts

Further theme. Context-dependent support systems

Further theme. Communicating permission for health behaviour

Theme 3. Identified priority health-protective behaviours

Subtheme 3a. Eating and drinking

Subtheme 3b. Rest

Subtheme 3c. Toileting

Subtheme 3d. Psychological support

**Table 2a.**

*Tabulated EHBM domains of demographic variables and psychological factors and contextual additional factors within the interviews*

| **Domain** | **Themes** | **Sub-themes** | **Further Themes** |
| --- | --- | --- | --- |
| **Demographic Variables** | Age | Physical capability | - - - Capacity to cope with neglect of health needs |
|  |  | Maturity of perspective | - - - Expectations     - Resilience     - Difference in perspective |
|  | Sex | Capacity | - - - Sex-specific health behaviours     - Sex-specific physical needs |
|  |  | Sex differences | - - - Within-specialty sex dominance     - Gender stereotyping and discrimination |
|  | Ethnicity | Identity | - - - Belonging     - Values and beliefs |
|  |  | Integration and inclusion | - - - Country of working residence     - Cultural norms and expectations |
|  | Occupational status | Professional status & occupational responsibilities | - - - Job description determines self-efficacy & creates health behavior disparities     - Specialism predicts the demands competing with personal health |
|  |  | Job responsibilities | - - - Role predicts level of competing demands |
|  | Family status | Dependent commitments | - - - Impact of dependents on shift work     - Employment inflexibility     - Carer responsibilities |
|  |  | Off-shift recovery | - - - Ability to rest off-shift     - Pressure     - Support |

| **Domain** | **Themes** | **Sub-themes** | **Further Themes** |
| --- | --- | --- | --- |
| **Psychological Characteristics** | Personality | Maladaptive perfectionism | - - - Vulnerability to burnout |
|  |  | Social vulnerability | - - - Psychosocial pressure     - Imposter syndrome |
|  | Bi-directional trait effects associated with high self-expectation | Ambition | - - - Diligence & work ethic     - Perceived success and pride     - Adaptive strategies to cope |
|  |  | Negative trait effects | - - - Perceived failure     - Self-critical |
|  | Mental health | Anxiety & depression | - - - Vulnerability to burnout |
|  |  | Emotional competence | - - - Anxious avoidant     - Responses to stress & trauma |
|  | | |  |
| **Further Contributing Predisposing Contextual Factors** | Systemic position | Unexpected challenges | - - - Political climate     - Viral risk to population     - Government policies |
|  |  | Unstable organization | - - - NHS funding distribution     - Appraisal & 360-degree feedback |
|  | Work context | Departmental variances | - - - Acute vs chronic wards     - Within vs without team working     - Perceived systemic alliance |
|  |  | Compromised working conditions | - - - Staffing     - Adequacy of resources     - The work is never 'done' |

**Table 2b.**

*Tabulated EHBM domain of health motivation and sub-domains of perceived susceptibility, severity, benefits, and barriers, in addition to contextual additional factors within the interviews*

| **Domain: Health Motivation** | **Sub-domains** | **Themes** | **Sub-themes** | **Further Themes** |
| --- | --- | --- | --- | --- |
|  | **Perceived Susceptibility** | Low autonomy | Professional responsibility | - - - - Acceptance of expected self-sacrifices       - Letting team down       - Unhealthy senior role models |
|  |  |  | Disempowering pressure | - - - - Fear of non-conformity       - Unable to abandon ward |
|  |  | Minimization of vulnerability | Reprioritization | - - - - The patient’s ill health supersedes personal health |
|  |  |  | Needs suppression | - - - - Age is viewed as a buffer to vulnerability       - Complacency       - Health needs as a perceived weakness       - Denial of symptoms       - Physical & emotional dissociation |
|  |  | | |  |
|  | **Perceived Severity** | Personal impact | Behavioural gravity | - - - - Risk taking & self-sacrifice       - Self-criticism       - Underestimation of needs |
|  |  |  | Consequences | - - - - Concern from loved ones       - Cumulative impact       - Impact on health |
|  |  | Work performance | Risk to patients | - - - - Inaccurate risk appraisal       - Impact of impaired decision making on patient care |
|  |  |  | Risk to professional integrity | - - - - Avoiding decision-making when health is compromised       - Being present is more important than being effective       - Guilt from medical judgement error |
|  |  | Occupational norms | Psychosocial collusion | - - - - Senior doctors minimize the impact on junior doctors       - Lack of support       - Ethical judgement |
|  |  |  | Autonomy | - - - - Permission to attend to needs |
|  |  | | |  |
|  | **Perceived Benefits** | Fitness to practice | Competence | - - - - Decision making, performance & patient safety |
|  |  |  | Staff retention | - - - - Shift endurance       - Longevity |
|  |  | Improved work ambiance | Normative shifts | - - - - Healthier standards       - Reduced professional dissonance |
|  |  |  | Positive milieu | - - - - Modelling positive health behavior       - Staff safety       - Consideration of others needs |
|  |  | Well-being | Physical health benefits | - - - - Lifestyle       - Reduced personal health risk |
|  |  |  | Psychological health benefits | - - - - Buffer to stress       - Improved Cognition |
|  |  | | |  |
|  | **Perceived Barriers** | Occupational beliefs & perceptions | Collective norms | - - - Unrealistic expectations     - Expected demands |
|  |  |  | Systemic groupthink | - - - Systemic culture rewards selflessness     - Work commitments take priority |
|  |  | Physical and procedural barriers | COVID-19 related protocols | - - - PPE |
|  |  |  | Understaffing | - - - No staff to enable relief cover |
|  |  |  | COVID-19 related protocols | - - - PPE |
|  |  |  | Understaffing | - - - - No staff to enable relief cover |
|  |  |  | Health resource accessibility | - - - Access to food & drink       - NHS regulations & policies |
|  |  | Workload | Principled obligations | - - - - Demands on the ward       - Ethical responsibility |
|  |  |  | Time pressure | - - - - Time-sensitive tests and treatment       - Time stress |
|  |  | | |  |
|  | **Further Psycho-socio-systemic Mediators** | Health service operational culture | Critical work ethos | - - - Judgement from colleagues |
|  |  |  | Permeated role expectancies | - - - Professional & personal dissonance     - Appraisal of the payoff of health sacrifice     - Fear of senior doctors |
|  |  |  | Disenchantment | - - - Non-compliance to policies and regulations     - No faith in systemic policy rationale |
|  |  | Governance | Disconnected understanding | - - - Regulatory priorities     - Disengaged from policy outcome     - Tokenistic & ineffective attempts at health promotion |
|  |  |  | Misaligned strategic objectives | - - - Policy implementation strategy     - Disconnect from the needs of frontline staff     - Systems do not support staff health |

**Table 2c.**

*Tabulated EHBM domains of self-efficacy, cues to action and action*

| **Domain** | **Themes** | **Sub-themes** | **Further themes** |
| --- | --- | --- | --- |
| **Self-efficacy** | Diminished self-efficacy | Time constraints | - - - - Workload       - Absence of relief medic |
|  |  | Reactive milieu | - - - - Patient emergencies       - Ward staffing requirements       - Stress & burnout |
|  |  | Unable to overcome obstacles to health-protective behaviour | - - - - PPE removal risks infection       - Fear of judgement       - Food & drink are unavailable       - Night shift |
|  | Enhanced self-efficacy | Self-assured senior authority | - - - - Increased time off the ward       - Self-management of time       - Less concerned about visible performance |
|  |  | Confidence in health-related prioritization | - - - - Self-preservation       - Autonomy |
|  |  |  |  |
| **Domain** | **Themes** | **Sub-themes** | **Further themes** |
| **Cues to action** | Extrinsic prompts | Operational staff prompts | - - - - Top-down modelling       - Culture shift       - Health education       - Senior doctors monitoring staff needs       - Formal cues       - Systems to cover patient caseload |
|  |  | Strategic stimulus | - - - - Clinical guiding principles       - Systemic expectations       - Policy dissemination       - Monitoring audits |
|  | Intrinsic cues | Perceptual | - - - - Self-awareness       - Understanding of psychophysiological impact       - Cognizance of diminished wellbeing on patient care |
|  |  | Activating and provoking | - - - - Self-motivation       - Personal responsibility-taking       - Anticipatory regret |
|  |  | Cognitive-behavioural | - - - - Planned behavioural intent       - Self-directed prompts       - Reactive homeostatic behavior |
|  | | | |
| **Domain** | **Themes** | **Sub-themes** | **Further themes** |
| **Action** | Individual activation | Proactive personal initiation | - - - Planned behavioural action |
|  |  | Reactive personal activation | - - - Behavioral reaction to needs     - Actions responsive to individual health & work context applied |
|  |  | Self-advocacy | - - - Deviation from maladaptive social norms |
|  | Adherence to systemic policies & procedures | System-wide directives | - - - Monitoring and reporting     - Explicit implemented support     - Regulatory implementation |
|  |  | Operational policy execution | - - - Top-down prompts     - Visual health promotion prompts     - Context dependent support systems     - Communicating permission for health behaviour |
|  | Identified priority behaviors | Health-protective behaviors | - - - Eating & drinking     - Rest     - Toileting     - Psychological support |
