# Supplementary material for: “Are you OK doctor?” An expanded health belief model exploration of doctors’ experiences and perspectives of on-shift health behaviour
Source: Int J Qual Stud Health Well-being. 2024 Aug 5;19(1):2388795. doi: 10.1080/17482631.2024.2388795 (PMC11305022; doi:10.1080/17482631.2024.2388795)
Supplement: Supplementary File 1 Research Protocol.docx [file ZQHW_A_2388795_SM2514.docx]

**Supplementary File 1.**

*Research Protocol*

**Interview Schedule**

The below interview schedule provides an indicative semi-structured outline for the focus group interviews (FGIs) and individual depth interviews (IDIs).

The schedule is developed based upon the Expanded Health Belief Model (EHBM) developed by Rosenstock et al., (1988) to understand intention to change health behaviour and maintains that health behaviour change is based upon a rational cognitive appraisal (Munro et al., 2007) which can inform change processing. All EHBM elements are included in the qualitative inquiry with the exception of ‘Demographic Variables’ as a contributing factor; these contain potentially identifiable information, so this information will be sought via the secure online survey.

Questions are not listed in the order of the EHBM to reflect a funnelling effect and centre the more challenging depth of content, therefore ensuring participants conclude on a lighter construct. Due to the potential crossover in responses, inquiry regarding self-efficacy and perceived barriers have been separated to enable distinct reflection pre- and post- consideration of the perceived severity of neglecting the on-shift health behaviours.

**Part 1. Briefing**

- Welcome Participants
- Researcher introduction
- Clarify aim
- Explain the purpose of an FGI/IDI
- Review consent, recording, anonymity and confidentiality
- Indicate the time parameters of up to 60 minutes for the IDI and up to 90 minutes for the FGI.
- Invite questions

**Part 2. Semi-structured Indicative Interview Questions**

Broad questions and probes based upon the contextual application of the EHBM constructs are designed to explore key concepts. Questions are semi-structured, however, the use of EHBM terminology will not be paraphrased, it will remain true to the model across all FGIs and IDIs to ensure consistency and minimise interpretative variation.

***2.1 Context***

**Objective**: To establish a shared understanding of terminology and identify prioritisation of needs through dominance and sequence of each behaviour.

**Question 2.1.1**: What health protective self-care behaviours are a component of your on-shift working practice?

*Probe A*: Hydration specific inquiry if not acknowledged in the responses

***2.2 EHBM Contributing Factor: Psychological characteristics***

**Objective**: To explore socio-occupational identity

**Question 2.2.1**: As you’re aware, this study is specifically focussed on exploring perceptions of on-shift health protective behaviours and perceived effects on competence. Are there any common **psychological characteristics** you have noticed among doctors?

*Probe A*: Are these **psychological characteristics** likely to impact upon on-shift health behaviours? If so, how?

*Probe B*: Which health behaviours are most likely to be affected by these **psychological characteristics**?

-------------------- Brief Transition Summary--------------------

***2.3 EHBM Construct: Perceived Benefits***

**Theoretical Rationale**: The EHBM predicts that **perceived benefits** (e.g., health, augmenting competence) will influence behavioural action.

**Objective**: To explore participant perceptions of the benefits of on-shift health protective behaviours.

**Question 2.3.1**: What, if any, do you **perceive** as being the **benefits** to undertaking the on-shift health protective behaviours you have identified?

*Probe A*: What, if any, do you **perceive** the personal **benefits** to be?

*Probe B*: What, if any, do you **perceive** the professional competence **benefits**/ benefits to patients to be?

*Probe C*: Has COVID-19 and associated workplace protocols affected your **perception** of the **benefits** of attending to your own on-shift health needs? If so, how?

*Probe D*: Hydration specific effects if not acknowledged

-------------------- Brief Transition Summary--------------------

***2.4 EHBM Proximal Action Factor: Self-efficacy***

**Theoretical Rationale**: The EHBM considers perceived **self-efficacy** as a proximal factor of action.

**Objective**: To explore participant perceptions of their **self-efficacy** i.e., the perception of their capability to succeed at improving their on-shift health protective behaviours.

**Question 2.4.1**: To what extent, if any, do you feel you have **self-efficacy** to respond to your on-shift health needs?

*Probe A*: What, affects your perceptions of **self-efficacy**?

*Probe B*: Has COVID-19 and associated workplace protocols affected your **perceived** self-efficacy to respond own on-shift health needs? If so, how?

*Probe C*: Hydration specific effects if not acknowledged

-------------------- Brief Transition Summary--------------------

***2.5 EHBM Construct: Perceived Susceptibility***

**Theoretical Rationale**: The EHBM predicts that higher **perceived susceptibility** results in a higher likelihood of engagement in health-protective behaviour.

**Objective**: To explore participant perceptions of their own susceptibility to neglecting their on-shift health protective behaviours.

**Question 2.5.1**: Do you **perceive** yourself as being **susceptible** to neglecting your on-shift health protective behaviours?

*Probe A*: What context or circumstances do you **perceive** as increasing your **susceptibility** to neglecting your on-shift health protective behaviours?

*Probe B*: Do you **perceive** any specific health protective behaviours as being more **susceptible** to diminished self-care in the circumstances you have highlighted?

*Probe C*: Has COVID-19 and associated workplace protocols affected your **perception** of the **susceptibility** to neglecting your own on-shift health needs? If so, how?

*Probe D*: Hydration specific effects if not acknowledged

-------------------- Brief Transition Summary--------------------

***2.6 EHBM Construct: Perceived Severity***

**Theoretical Rationale**: The EHBM predicts that higher **perceived severity** from the appraisal of knowledge of the consequences results in a higher likelihood of engagement in health-protective behaviour.

**Objective**: To explore participant **perceptions** of the **severity** of the consequences of neglecting their on-shift health protective behaviours.

**Question 2.6.1**: How **serious** do you **perceive** the effects to be of poor attention to your health needs? and what impact would this have on my work?

*Probe A*: How **severe** would the consequences be to your wellbeing?

*Probe B*: How **severe** would the consequences or impact be to your professional competence?

*Probe C*: Would neglecting some health behaviours have more **severe** consequences than others? How do you perceive the **priority** order?

*Probe D*: Has COVID-19 and associated workplace protocols affected your **perception** of the **severity** of neglecting your own on-shift health needs? If so, how?

*Probe E*: Hydration specific effects if not acknowledged

-------------------- Brief Transition Summary--------------------

***2.7 EHBM Construct: Perceived Barriers***

**Theoretical Rationale**: The EHBM proposes that health-protective behaviours are affected by **perceived barriers** to taking action based upon appraisal of any obstacles to change (e.g., PPE, time).

**Objective**: To explore participant **perceptions** of the **barriers** to attending to their on-shift health protective behaviours.

**Question 2.7.1**: Are there any barriers that you perceive to attending to your health needs on shift?

*Probe A*: Do some health behaviours have greater barriers than others?

*Probe B*: Has COVID-19 and associated workplace protocols affected these **barriers**? If so, how?

*Probe C*: Hydration specific effects if not acknowledged

-------------------- Brief Transition Summary--------------------

***2.8 EHBM Construct: Cues to Action***

**Theoretical Rationale**: According to the EHBM a **cue to action** is necessary to prompt engagement in any health protective behaviour change. These cues may be intrinsic (e.g., beliefs or benefits) or extrinsic (e.g., socio-systemic pressure to engage in behaviour conducive to augmenting competent practice).

**Objective**: (1) To examine perceptions of current intrinsic and extrinsic **cues to action** that prompt on-shift health protective behaviours. (2) To explore potential **cues to action** that may augment on-shift health protective behaviours.

**Question 2.8.1**: What **cues to action**, or prompts, do you **perceive** as currently facilitating your on-shift health protective behaviours.

*Probe A*: Do some health behaviours have different **cues to action** than others?

*Probe B*: Are there any intrinsic **cues to action** (e.g., beliefs or benefits) that currently facilitate your on-shift health protective behaviours?

*Probe C*: Are there any extrinsic (e.g., socio-systemic) **cues to action** that currently facilitate your on-shift health protective behaviours.

*Probe D*: Has COVID-19 and associated workplace protocols affected these **cues to action**? If so, how?

*Probe E*: Hydration specific effects if not acknowledged

**Question 2.8.2**: What **cues to action**, or prompts, do you **perceive** as having potential to facilitate your on-shift health protective behaviours in the future.

*Probe A*: Do some health behaviours have different **cues to action** than others?

*Probe B*: Are there any intrinsic **cues to action** (e.g., beliefs or benefits) that could facilitate your on-shift health protective behaviours in the future?

*Probe C*: Are there any extrinsic (e.g., socio-systemic) **cues to action** that could facilitate your on-shift health protective behaviours in the future?

*Probe D*: Hydration specific ideas if not acknowledged

--------------------Brief Transition Summary--------------------

**Part 3. Debriefing**

- Summarise a brief overview of the discussion
- Invite any outstanding comments or questions
- Describe the next steps for the study
- Normalise and highlight the sources of available follow up support
- Reiterate option for individual debriefing and any further guidance on self-referral support options
- Thank participants for their time and sharing their experiences

**Ethical Considerations Prior to Study Initiation**

This study will recognise the duty of care to participants and institutions affiliated with this research. Maintaining integrity and respectful practice with a strong awareness of legal and moral rights will be ensured by compliance with the law, upholding scientific standards and avoiding personal or professional harm. This study involves human research. Therefore, ethics approval would be obtained from the University's School Level Research Ethics Committee (SREC) prior to the commencement of any research. Although participants will be NHS employees, they will not be recruited directly from the NHS, nor will their participation procedurally impact upon their professional work and none of the procedures will take place at NHS sites; therefore, NRES approval is not required.  Therefore, the following considerations will be addressed prior to study initiation:

1. Ethical approval will be obtained prior to study initiation.
2. Informed consent will be provided via the provision of initials.
3. Participants will provide their email addresses to enable communication and coordination of interview appointments*.* Researchers will not have any direct face-to-face contact with participants; contact will be maintained via email. However, a telephone number will be required for optional telephone briefing and debriefing appointments (at the participant's request).
4. This study has a transparent aim. This study's objectives will always be explicit to participants and the advertising organisations.
5. The researcher's email and senior supervisor's contact details will be provided so that participants can raise any concerns that may arise during the study.
6. All data will be stored in a password-protected file, and all conceivable steps will be taken to ensure its security.
7. Whilst the study procedures are not designed to elicit distressing experiences, exploratory questions about health and competence may potentially trigger reflective discontent. This will be supported by debriefing information and links to further support services.
8. Participant time is a consideration; participant time commitment has been managed by minimising the survey length and the FGI/Interview duration undertaken single timepoint to ensure minimal impact.
9. Potential participants will be reassured that their engagement is entirely voluntary, no explanation of refusal is required, and there will be no penalty for non-participation. Later withdrawal of data will be possible for up to a month following completion to enable specific extraction prior to data analysis and potential publication of data.
10. Participants may omit any questions they are uncomfortable answering; their right to do so will be explicit.
11. No direct risks to the researchers have been identified due to the remote design of this study; telephone contact will be via a withheld number using an office phone.
12. The participant briefing will include recommendations to support their anonymity/confidentiality on video call, this will include the option to turn their video off and recommendations to ensure they participate in privacy (e.g., by setting their profile name as their participant I.D.), and ensuring that they are not overheard by others in the household (e.g., by wearing headphones).
13. Video calls will be recorded and erased following anonymised transcription.
14. This study acknowledges that the rights of participants to confidentiality is paramount. Therefore, safe and secure storage of data will be prioritised to ensure ethical practice by utilising a password-protected electronic filing system. Survey software (OS) will be used under the University licence and the software is GDPR compliant. Data Protection legislation will be adhered to.

The researchers declare that there is no conflict of interest. This study will adhere to the guidelines dictated by The British Psychological Society's Code of Human Research Ethics (2021).

**The Transcription and Anonymization of Qualitative Data**

***Study***

“Are you OK doctor?” A Health Belief Model exploration of doctors’ experiences and perspectives of on-shift health behavior

***Aim***

The orthographic transcription methods aim to present a complete and clear rendering of the interviews, and to tabulate for further coding and analysis. This study adopted a transparent, reproducible, and rigorous approach to the screening and anonymization of the qualitative data to protect the identification of participants but preserve the integrity of the responses and communication of salient themes.

***Methods***

This protocol was informed by the recommendations on qualitative data anonymization made by Braun and Clarke (2022), and Saunders et al. (2015). Transcripts were manually corrected for Teams software transcription errors. Timestamping was retained. These orthographic transcripts include all verbal utterances from all speakers; this includes both semantic sounds e.g., um, er, and actual words. There was no correction of content into ‘standard’ English, or removal of slang. Sentence structure errors were not corrected. Omission errors were not corrected. (.) was used to indicate short pauses, (...) was used to indicate longer pauses.

Qualitative transcript responses were screened by the researcher to identify specific anonymization criteria specific to the sample. Further collaborative discussion of each identified anonymity concern was addressed through consultation between each of the researchers to balance confidentiality with the preservation of content and themes.

The preliminary anonymity criteria identified and details of how each of these will be addressed are as follows:

1. **People’s names**

In all cases these will be substituted with their generic title e.g., Dr, Professor. No pseudonyms will be used, participants will instead be differentiated by their allocated participant identifier number (I.D.).

1. **Locations, hospitals, and specialized departments**

References to broad specialties (e.g., cardiology) and roles (e.g., junior doctor) was retained, specialized small sub-departmental structures will be removed to preserve the anonymity of the survey respondents.

1. **Specific projects and training references**

Named specific projects and training programs was either removed or neutralized as ‘project’ or ‘training’.

1. **Highly specialized occupations**

In some cases, highly specialized titles was genericized to preserve salient meaning but protect the anonymity of any specific individual.

1. **Occupational relationships**

Where possible the relationships described was preserved with the anonymization of all identifiable details.

1. **Further identifiable information**

This included specialist professional interests unique to individual doctors.

The anonymization protocol was applied, and where anonymization was applied the reason for anonymization will appear in [*AR: Code*].

| **Anonymisation Reason** | **AR Code** |
| --- | --- |
| People’s names | AR: 1 |
| Locations, hospitals, and specialized departments | AR: 2 |
| Specific projects and training references | AR: 3 |
| Highly specialized occupations | AR: 4 |
| Occupational relationships | AR: 5 |
| Further identifiable information | AR: 6 |

***Handling Errors***

Errors related to sentence structure, and omission errors were not corrected in this process. However, definitions of colloquial terms and abbreviations were itemized and added to a supplementary file.

***Outcome***

Two versions of the qualitative transcript were developed:

1. An original un-anonymized version.
2. An unmarked transcription screened and edited for anonymity.

All video and transcript data will be stored in password-protected secure electronic files.

**Recommendations**

Due to the anticipated size of the sample in this study, cumulative effects may be broadly accounted for. This protocol recommends further anonymization for any specific individual-case-by-case analyses planned in this study.

**Reflexive Analysis Protocol**

This protocol outlines the reflexive analysis protocol for the study: “Are you OK doctor?” A Health Belief Model exploration of doctors’ experiences and perspectives of on-shift health behaviour.

**Table 1**

*The ‘Six-Phase’ Application of Braun and Clarke’s (2022) Thematic Analysis*

| **Analysis Phase** | **Methodology** |
| --- | --- |
| **Phase 1: Familiarization** | In phase one, the recorded video interviews were frequently reviewed to ensure the accuracy of the data corpus by editing the automated transcription. Following the transcription and anonymization protocol also supported familiarization. The transcribed IDIs and FGIs were tabulated, and analyzed through reading and active recusant immersion techniques on each data item. Notes were compiled to record reflections during this initial stage, guided by Braun and Clarke’s (2012) ‘suggested questions’. |
| **Phase 2: Generating initial codes** | Braun et al.’s (2019) “using the codes as building blocks” (p.855) approach was employed to find commonality among the codes or some shared patterned meaning across the dataset. Preliminary analysis notes were used to inform further structured data extraction and candidate coding. Initial themes were generated from the preliminary codes and established in an Excel file. Despite varied subjective experiences from this non-homogenous sample, many insights shared meaning. Clusters of meaning were organized based on the research objectives and inductive emergent codes from the open survey and interviews. Initial coding was based deductively on the EHBM, with an inductive cyclical approach thereafter. |
| **Phase 3: Searching and development of themes** | All researchers reviewed candidate themes for objective clarity and guidance to mitigate subjectivity. The whole dataset was repeatedly revisited while simultaneously reviewing and mapping codes and themes. Quotes from the interviews supporting or refuting each were organized and categorized, and preliminary thematic mapping of initial coding frameworks was undertaken. |
| **Phase 4: Reviewing themes** | Developed thematic mapping using reflexive grouping of codes and themes assisted in visually understanding the connections between doctors’ similar subjective experiences and highlighting any inconsistencies and duplications. All researchers engaged in an ongoing reflexive triangulation dialogue regarding the recursive interpretive analytic process and reviewed final themes and codes to enhance concordance and confirmability (Nowell et al., 2017). |
| **Phase 5: Refining, defining, and naming themes** | Through assessment of heterogeneity and homogeneity, themes were reviewed and refined to ensure their ‘central organizing concept’ (Braun et al., 2019). Linguistics were assessed for the objectivity of interpretation, reflectivity of terms, and labelling of codes and themes. |
| **Phase 6:**  **Final write-up** | At this integral phase (Braun & Clarke, 2012), the analysis linked and refined the five stages by corroborating participant quotes with reference to the research aims. More latent connections also became apparent to ensure the themes represented the data. Findings were then contextualized within the literature and extracts that captured key analytic essence were embedded to illustrate the analytic narrative. |

**Analysis Approaches**

***Deductive thematic analysis***

Predetermined analysis included: the Expanded Health Belief Model (EHBM) domains within a pre-existing theory-driven coding frame. Furthermore, to address the research aims, thematic mapping of the contemporary NHS landscape including the impact of COVID-19 and perceptions of health behaviour, competence and impact on patient safety were examined. The deductive analysis provided a preliminary categorisation framework, but inductive coding was subsequently used to explore experiences.

***Inductive thematic analysis***

Data-driven retrospective (post-hoc) research questions that informed inductive coding and analysis included:

- How and why is the health impact different at various occupational grades and specialties?
- What demographic variables affect health and competence effects, and how?
- Are there consistent barriers and facilitators to on-shift health-protective behaviours?
- What realistic interventions would facilitate health protective behaviour.

**Further Reflexive Procedures**

Engagement with literature relevant to the analytic themes was undertaken on completion to avoid narrowing and biasing the analytic view as advocated by Braun and Clark (2006).

**References**

Braun, V., & Clarke, V. (2006). Using thematic analysis in psychology. Qualitative Research in Psychology, 3(2), 77–101. [https://doi.org/10.1191/1478088706qp063oa](https://psycnet.apa.org/doi/10.1191/1478088706qp063oa)

Braun, V., & Clarke, V. (2012). *Thematic Analysis*. American Psychological Association.

Braun, V., & Clarke, V. (2019). Reflecting on reflexive thematic analysis. *Qualitative Research in Sport, Exercise and Health*, *11*(4), 589-597. <https://doi.org/10.1080/2159676X.2019.1628806>

Braun, V., & Clarke, V. (2021). Can I use TA? Should I use TA? Should I not use TA? Comparing reflexive thematic analysis and other pattern‐based qualitative analytic approaches. *Counselling and Psychotherapy Research*, *21*(1), 37-47.

Braun, V., & Clarke, V. (2022). Conceptual and design thinking for thematic analysis. Qualitative Psychology, 9(1), 3–26. [https://doi.org/10.1037/qup0000196](https://psycnet.apa.org/doi/10.1037/qup0000196)

Munro, S., Lewin, S., Swart, T., & Volmink, J. (2007). A review of health behaviour theories: how useful are these for developing interventions to promote long-term medication adherence for TB and HIV/AIDS? *BMC Public Health*, (*7*):104. doi: 10.1186/1471-2458-7-104

Nowell, L. S., Norris, J. M., White, D. E., & Moules, N. J. (2017). Thematic Analysis: Striving to Meet the Trustworthiness Criteria. *International Journal of Qualitative Methods*, *16*(1). <https://doi.org/10.1177/1609406917733847>

Oates, J., Carpenter, D., Fisher, M., Goodson, S., Hannah, B., Kwiatowski, R., Prutton, K., Reeves, D., & Wainwright, T. (2021). BPS Code of Human Research Ethics. British Psychological Society. https://www.bps.org.uk/guideline/bps-code-human-research-ethics

Rosenstock, I. M., Strecher, V. J., & Becker, M. H. (1988). Social learning theory and the Health Belief Model. *Health Education Quarterly*, *15*(2), 175–183. <https://doi.org/10.1177/109019818801500203>

Saunders, B., Kitzinger, J., & Kitzinger, C. (2015). Anonymising interview data: challenges and compromise in practice. *Qualitative Research*, *15*(5), 616–632. <https://doi.org/10.1177/1468794114550439>
